# Hemiarthroplasty with a long femoral stem and proximal cement augmentation for a myeloma-related pathological femoral neck fracture: a case report

**DOI:** 10.3389/fsurg.2026.1819823

**Published:** 2026-10-28

**Authors:** Zhen Shang, Changkun Wu, Jianyong Liu

**Affiliations:** Department of Sports Medicine, Weifang People’s Hospital, Shandong Second Medical University, Weifang, China

**Keywords:** femoral neck fracture, hemiarthroplasty, joint reconstruction, multiple myeloma, pathological fracture

## Abstract

**Background:**

Pathological femoral neck fractures are a frequent skeletal complication of multiple myeloma due to extensive osteolytic bone destruction. However, extensive proximal femoral invasion with massive cavitary destruction leading to femoral neck fracture is clinically uncommon, and optimal surgical management remains challenging because of poor bone quality and high mechanical failure risk.

**Case presentation:**

We report a patient with a known history of multiple myeloma who sustained a low-energy fall resulting in a pathological fracture of the right femoral neck. Imaging demonstrated diffuse osteolytic involvement of the pelvis and proximal femur with severe osteoporosis and degenerative hip changes. Given the patient's poor general condition and multiple comorbidities, a multidisciplinary team (MDT) discussion involving orthopaedics, hematology, cardiology, endocrinology, and neurology was conducted to formulate an individualized perioperative strategy. Bipolar hemiarthroplasty was performed using a long femoral stem with distal press-fit fixation, combined with debridement of tumor-infiltrated tissue, proximal cement augmentation of osteolytic bone defects, and supplemental plate and cerclage cable fixation. This reconstructive strategy was selected as an alternative to proximal femoral replacement to achieve immediate mechanical stability and facilitate early mobilization. Intraoperative autologous blood salvage and reinfusion were used as part of the perioperative blood-management strategy, with no immediate procedure-related complications observed.

**Results:**

The patient was followed for 15 months after surgery. At the latest follow-up, she remained ambulatory with satisfactory hip function and a Harris Hip Score of 84 points. Follow-up radiographs demonstrated stable reconstruction without prosthetic loosening, dislocation, periprosthetic fracture, or other major implant-related complications.

**Conclusion:**

Hemiarthroplasty with a long femoral stem, proximal cement augmentation, and supplemental fixation may represent a viable option for selected patients with multiple myeloma-related pathological femoral neck fractures and extensive osteolytic destruction. Careful assessment of bone loss and individualized multidisciplinary planning are important for balancing mechanical stability and perioperative risk.

## Introduction

Multiple myeloma is frequently associated with extensive osteolytic bone disease, predisposing patients to pathological fractures even after minimal trauma (1). The proximal femur is a common site of involvement, and femoral neck fractures in this setting can result in sudden loss of ambulation and substantial morbidity (2, 3). Surgical treatment is often required to restore mobility, relieve pain, and provide mechanical stability (4, 5).

Femoral neck fractures secondary to multiple myeloma present challenges distinct from those encountered in routine osteoporotic fractures (6). Tumor-related osteolysis, cortical thinning, and cavitary bone defects may substantially compromise proximal femoral bone stock, reducing the reliability of conventional internal fixation and increasing the risk of mechanical failure (7). In addition, anemia, metabolic bone impairment, systemic disease burden, and other medical comorbidities may further complicate perioperative management (8). Arthroplasty can provide immediate stability and pain relief, but implant selection, fixation strategy, and the extent of reconstruction must be individualized according to the remaining bone stock and local disease involvement (9). With improvements in systemic therapy and survival, durable reconstruction has also become increasingly important in patients with multiple myeloma (10).

Although osteolytic skeletal involvement is common in multiple myeloma, extensive proximal femoral destruction with pathological fracture represents a challenging reconstructive scenario. Surgical management should be individualized according to the extent of bone destruction, remaining femoral bone stock, systemic disease burden, and anticipated functional requirements (11). Such patients may also require multidisciplinary perioperative assessment because of concomitant systemic disease and medical comorbidities (12). This case report was prepared in accordance with the CARE reporting guidelines (13). We report a case of a multiple myeloma-related pathological femoral neck fracture with extensive proximal femoral osteolysis in which reliable internal fixation was not feasible. Hemiarthroplasty using a long femoral stem with distal press-fit fixation, proximal cement augmentation, and supplemental plate–cable fixation was therefore performed. This case highlights the importance of individualized reconstructive decision-making in the setting of severe myeloma-related bone loss.

## Case presentation

A 66-year-old woman with a 1.5-year history of multiple myeloma presented with acute right hip pain and inability to bear weight following a low-energy fall. Multiple myeloma had been diagnosed approximately 18 months before the current admission and was classified as ISS stage II and R-ISS stage II. The patient had not received standardized systemic anti-myeloma therapy before the current admission **(**Supplementary Table 1**)**. The patient was immediately unable to bear weight. The medical history was significant for long-standing type 2 diabetes mellitus, poorly controlled hypertension, and anemia. The myeloma had not been managed with standardized systemic therapy prior to admission. Physical examination revealed shortening and external rotation of the right lower limb with marked tenderness around the hip. Passive motion elicited severe pain. Distal neurovascular status was intact.

Plain radiographs and computed tomography demonstrated diffuse osteolytic lesions involving the pelvis and bilateral proximal femora, consistent with myeloma-related bone disease **(**Figure 1**)**. A pathological fracture of the right femoral neck was identified, accompanied by severe osteoporosis and degenerative changes of the hip joint. Given the extensive proximal femoral destruction and high risk of fixation failure, surgical treatment with joint replacement was planned after multidisciplinary discussion. The overall clinical course from the diagnosis of multiple myeloma to the 15-month postoperative follow-up is summarized in Supplementary Table 2.

**Figure 1:**
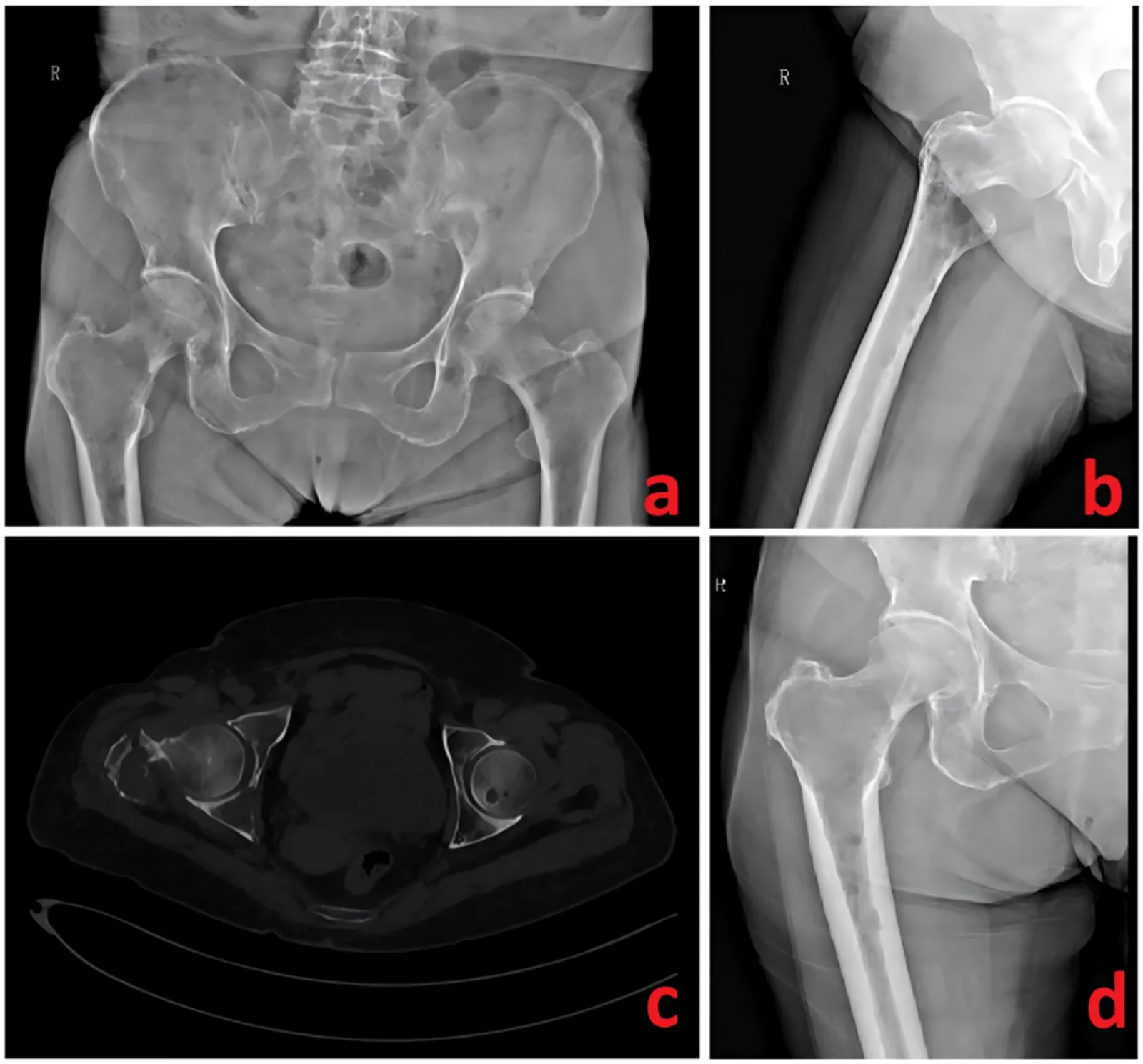
Preoperative imaging of the right hip. **(a)** Anteroposterior radiograph of the pelvis demonstrating diffuse osteolytic changes involving the pelvis and proximal femora. **(b)** Lateral radiograph of the right hip showing a pathological fracture of the femoral neck. **(c)** Axial computed tomography image revealing extensive osteolytic destruction of the proximal femur with cortical thinning and cavitary bone defects. **(d)** Anteroposterior radiograph of the right femur further illustrating compromised proximal femoral bone stock.

### Diagnostic assessment

Although the patient had a known history of multiple myeloma, the differential diagnosis of the destructive femoral neck lesion included a purely osteoporotic fracture, metastatic bone disease from another primary malignancy, and a primary bone tumor. A purely osteoporotic fracture was considered less likely because imaging demonstrated extensive multifocal osteolytic lesions, marked cortical thinning, and cavitary destruction involving the pelvis and bilateral proximal femora. The multifocal distribution of the lesions together with the established history of multiple myeloma also made an isolated primary bone tumor less likely. The underlying multiple myeloma was classified as ISS stage II and R-ISS stage II. During the current admission, the diagnostic workup included plain radiographs, CT, routine hematological and biochemical tests, and subsequent histopathological examination of the resected lesion. Laboratory testing showed a hemoglobin level of 106 g/L and a serum calcium level of 2.27 mmol/L. Histopathological examination of the proximal femoral tissue demonstrated plasma cell myeloma, with immunohistochemical findings supporting myeloma-related bone involvement.

### Surgical technique

The procedure was performed through a posterolateral approach under general anesthesia **(**Figure 2**)**. Intraoperatively, extensive tumor-related destruction involving the femoral neck, calcar, greater trochanter, and proximal femur was observed, with markedly thinned cortices and insufficient bone stock for reliable internal fixation. The femoral head was removed, and tumor-infiltrated bone and surrounding tissue were debrided and submitted for histopathological examination, which subsequently confirmed plasma cell myeloma.

**Figure 2:**
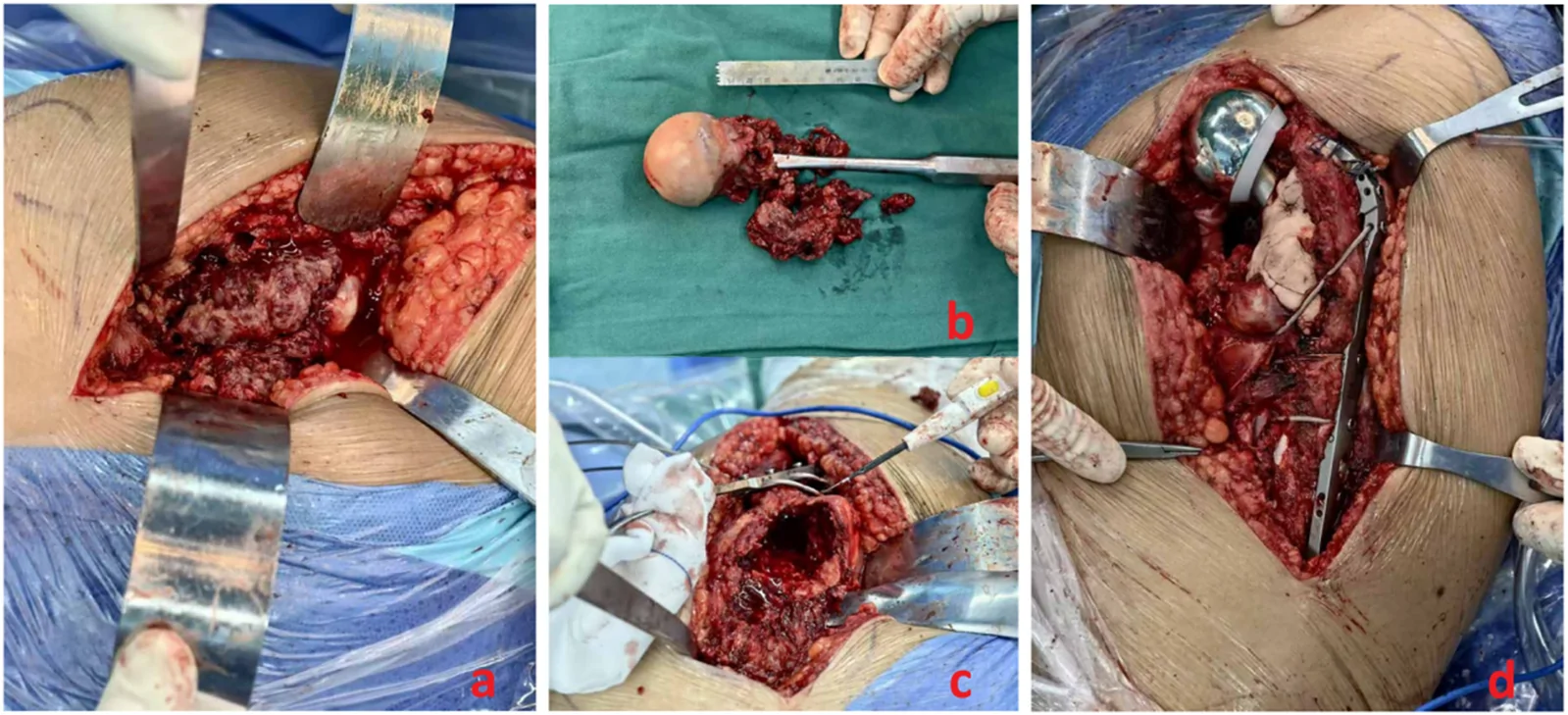
Intraoperative photographs demonstrating the surgical procedure. **(a)** Extensive tumor-infiltrated tissue and cavitary bone defects of the proximal femur exposed after capsulotomy. **(b)** Resected femoral head and surrounding tumor-infiltrated tissue following excision. **(c)** Large cavitary defect of the proximal femur after thorough debridement of tumor-involved bone. **(d)** Reconstruction of the proximal femur using a cemented long-stem hemiarthroplasty combined with supplemental fixation.

A long-stem biological type femoral component was selected to bypass the compromised proximal femur and achieve fixation in relatively preserved distal femoral bone. Bone cement was used to fill the extensive proximal osteolytic cavitary defects. Because of extensive weakening of the greater trochanter and proximal femur, a trochanteric extension plate combined with cerclage cables was used for supplemental mechanical stabilization. A bipolar hemiarthroplasty head was implanted. Intraoperative autologous blood salvage and reinfusion was used as part of the perioperative blood-management strategy.

### Outcome and follow-up

Postoperatively, the patient received prophylactic antibiotics, anticoagulation, and early rehabilitation. Wound healing was uneventful, and no early infection or implant-related complications were observed **(**Figure 3**)**. The patient experienced substantial pain relief and was able to ambulate with assistive devices before discharge. Histopathological examination confirmed bone involvement by plasma cell myeloma, and the patient was subsequently referred to the hematology service for further management of the underlying disease. After discharge, the patient received zoledronic acid (4 mg intravenously every 4 weeks) for the management of myeloma-related bone disease. Treatment adherence was satisfactory, and the therapy was well tolerated without clinically significant treatment-related adverse effects. The patient was followed for 15 months after surgery. At the latest follow-up, she remained ambulatory and maintained satisfactory hip function, with a Harris Hip Score of 84 points. Follow-up radiographs demonstrated stable positioning of the long-stem prosthesis and supplemental fixation, without evidence of prosthetic loosening, dislocation, or periprosthetic fracture. No major adverse, unexpected, or implant-related complications were observed during the follow-up period.

**Figure 3:**
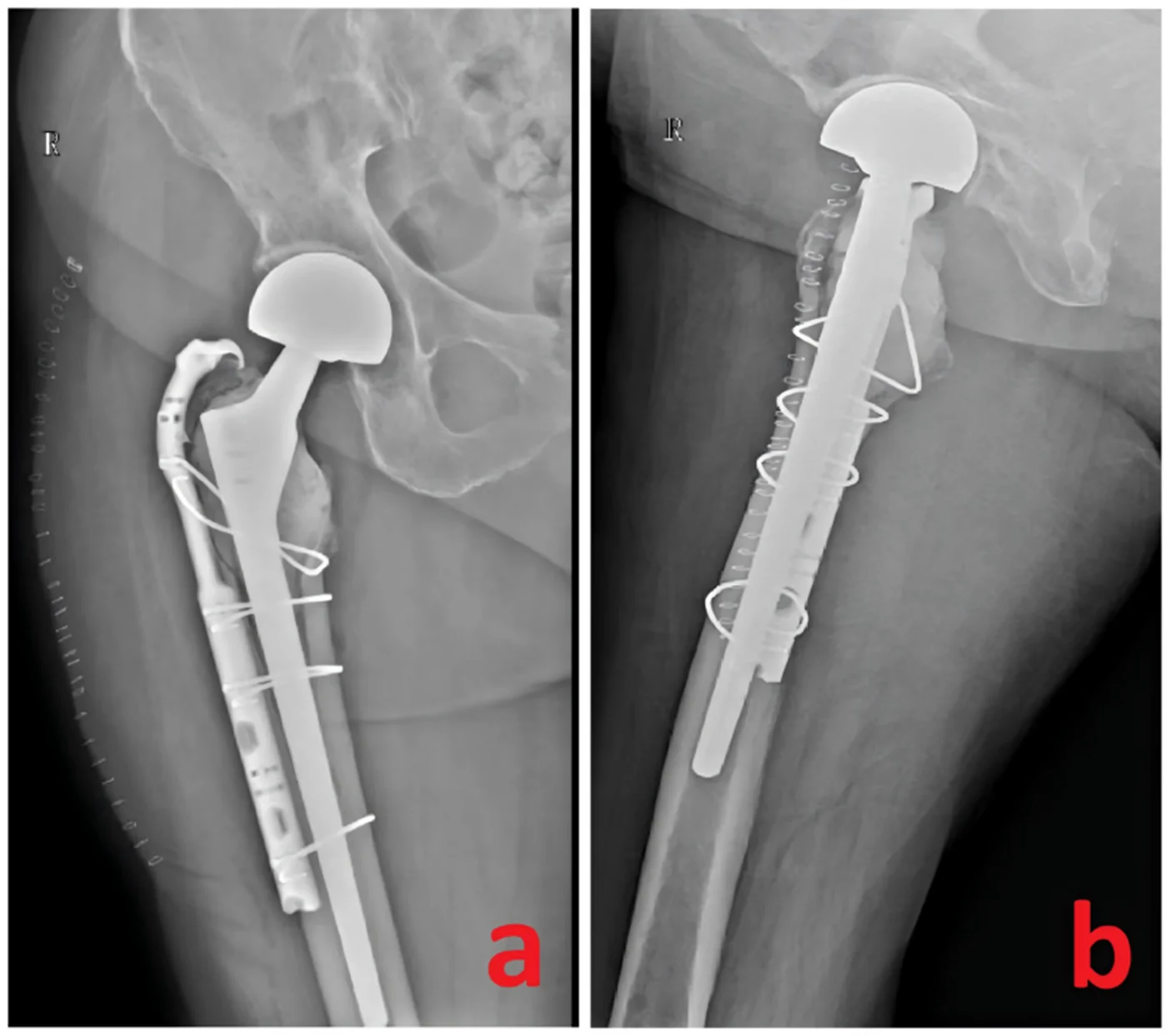
Postoperative radiographs of the right hip and femur. **(a)** Anteroposterior radiograph showing hemiarthroplasty with a long femoral stem and supplemental proximal femoral plate and cerclage cable fixation. Bone cement was used proximally to augment the osteolytic defects. **(b)** Lateral radiograph confirming appropriate implant alignment, stable fixation, and restoration of femoral continuity.

## Patient perspective

Before surgery, the patient reported severe right hip pain and inability to bear weight, which substantially limited her mobility and daily activities. Following surgery, she reported marked pain relief and progressive improvement in mobility. At the latest follow-up, she expressed satisfaction with the recovery of her walking ability and overall functional outcome.

## Discussion

Pathological femoral neck fractures in multiple myeloma represent a distinct clinical problem for arthroplasty surgeons (14). Unlike routine osteoporotic fractures, myeloma-related fractures occur in the setting of extensive osteolysis, compromised bone stock, and impaired bone remodeling (2). These factors substantially increase the risk of mechanical failure when internal fixation is attempted, particularly in the proximal femur, which is subjected to high biomechanical loads (5). Consequently, treatment strategies must prioritize immediate stability and mechanical durability.

In the present case, preoperative imaging and intraoperative findings demonstrated severe destruction of the proximal femur, including cortical thinning and large cavitary defects caused by tumor infiltration. These features precluded reliable internal fixation and favored joint replacement as the primary treatment strategy. Several surgical options may be considered for pathological fractures involving the proximal femur, including internal fixation, hemiarthroplasty, total hip arthroplasty (THA), and proximal femoral replacement or megaprosthetic reconstruction. Internal fixation may be appropriate when sufficient proximal femoral bone stock remains and reliable mechanical stabilization can be achieved; however, extensive osteolysis, cortical thinning, and cavitary destruction substantially increase the risk of fixation failure (5, 7). Hemiarthroplasty can provide immediate stability without acetabular reconstruction and may reduce operative time and dislocation risk in medically frail patients without substantial acetabular involvement. And THA may be preferred when clinically significant acetabular disease, pre-existing symptomatic arthropathy, or higher functional demands justify acetabular replacement (15). In contrast, proximal femoral replacement or megaprosthetic reconstruction is generally reserved for patients with extensive proximal femoral destruction in whom conventional femoral stem fixation cannot be reliably achieved, although these procedures are more extensive and may be associated with greater perioperative burden (16). The feasibility of hemiarthroplasty in complex femoral neck fracture scenarios has also been demonstrated by Najafi et al. (17), who reported two patients with neglected simultaneous bilateral femoral neck fractures treated with one-stage bilateral bipolar hemiarthroplasty. At 14 and 16 months of follow-up, the patients achieved Harris Hip Scores of 83 and 89, respectively, with satisfactory recovery of ambulatory function. Although the underlying etiology differed from that in the present case, these findings further support hemiarthroplasty as a reasonable reconstructive option in selected complex femoral neck fractures when treatment is individualized according to bone quality, acetabular involvement, patient condition, and functional requirements. In the present patient, extensive proximal femoral osteolysis and cavitary destruction made internal fixation unreliable. However, there was no major acetabular involvement requiring THA, and sufficient distal femoral bone remained to permit stable press-fit fixation with a long femoral stem. Proximal cement augmentation was used to support residual osteolytic defects. Therefore, long-stem biological type hemiarthroplasty with proximal defect augmentation was considered an appropriate reconstructive option that balanced mechanical stability with the need to minimize surgical burden in a medically complex patient. Recent reports relevant to the surgical management of complex pathological femoral neck and proximal femoral fractures are summarized in Table 1.

**Table 1:** Recent reports relevant to surgical management of complex pathological femoral neck and proximal femoral fractures.

Study	Clinical setting	Surgical management	Follow-up	Outcome/functional assessment
McCrosson et al., 2023 (22)	Solitary plasmacytoma presenting with a pathological femoral neck fracture	Distally fixing bipolar hemiarthroplasty	15 months	No postoperative complications; patient reported high satisfaction, good hip flexion, and no pain with log roll; formal functional score not reported
Small et al., 2023 (23)	Multiple myeloma with pathological femoral neck fracture and ipsilateral peritrochanteric/distal femoral lesions	THA with extended femoral stem, greater trochanteric claw plate, and supplemental fixation	3 months	Ambulated with a walker; substantial pain improvement; gradually returned toward usual daily activities; radiographs showed stable reconstruction
Najafi et al., 2024 (17)	Neglected simultaneous bilateral femoral neck fractures in two patients	One-stage bilateral bipolar hemiarthroplasty	14 and 16 months	No postoperative complications; Harris Hip Scores of 83 and 89, respectively; both patients recovered ambulatory function
Mahmoud et al., 2025 (5)	Multiple myeloma-associated displaced femoral neck fractures; 15 patients/16 hips	Hemiarthroplasty; predominantly cemented stems, including long stems in selected cases	Mean 18.2 months	Hemiarthroplasty considered a viable option; periprosthetic fracture 18.75%, heterotopic ossification 25%, acetabular erosion 6.25%; formal functional score not reported
Lesensky et al., 2025 (16)	Metastatic proximal femoral disease requiring endoprosthetic reconstruction	Proximal femoral replacement versus long cemented revision-stem hemiarthroplasty	Mean approximately 15–17 months	Long-stem hemiarthroplasty was associated with shorter operative time, less blood loss, faster progression to full weight-bearing, shorter hospitalization, and fewer postoperative complications

Additionally, The patient had anemia, diabetes, poorly controlled hypertension, and overall poor baseline status. Treatment planning therefore relied on MDT collaboration, with hematology guiding myeloma-related considerations and adjuvant therapy planning, cardiology assisting perioperative cardiovascular risk stratification, endocrinology supporting metabolic optimization (e.g., diabetes control and bone metabolism), and neurology contributing to perioperative neurological evaluation and rehabilitation readiness. This case illustrates the importance of MDT in achieving an individualized and safer surgical pathway for high-risk patients (12).

Substantial blood loss may occur during extensive tumor debridement and proximal femoral reconstruction. Previous studies of oncological proximal femoral reconstruction have reported considerable differences in estimated blood loss according to the reconstructive strategy (16). In the present case, intraoperative autologous blood salvage and reinfusion was used as part of the perioperative blood-management strategy, and no immediate complications attributable to the procedure were observed. However, concerns regarding potential tumor-cell contamination and oncological safety remain relevant in cancer surgery (18). Therefore, the experience in this single patient should not be interpreted as evidence of the general safety of autologous blood reinfusion in patients with multiple myeloma. Its use should remain individualized and guided by institutional blood-management protocols, and larger studies with dedicated oncological follow-up are required before broader conclusions can be drawn.

A long femoral stem was selected to bypass the severely osteolytic proximal bone and achieve primary mechanical stability through distal press-fit fixation in relatively preserved femoral diaphyseal bone (19). In this patient, sufficient distal bone stock allowed the use of an biological type long stem despite the compromised proximal femur. Bone cement was used to fill the residual proximal cavitary defects. Debridement of tumor-infiltrated bone and soft tissue facilitated reconstruction, although local tumor control also depends on systemic disease management. Longer follow-up is needed to assess the durability of stem fixation.

Supplemental plate and cable fixation was required to address extensive destruction of the greater trochanter and to reduce the risk of periprosthetic fracture (20), a recognized complication in patients with myeloma-related bone involvement (21). Restoration of proximal femoral continuity also facilitated abductor mechanism stabilization, which is important for postoperative hip stability and functional recovery. Although such adjunctive fixation increases surgical complexity, it may contribute to improved mechanical integrity in cases with severe bone loss.

With advances in systemic therapy, patients with multiple myeloma may achieve prolonged survival, shifting the surgical goal from temporary stabilization to durable reconstruction. At the same time, early functional recovery and pain control remain critical treatment goals, particularly in medically frail patients for whom rapid rehabilitation and reduced complication risk are prioritized. From a joint reconstruction perspective, treatment strategies must therefore balance oncologic considerations with long-term mechanical performance. This case suggests that hemiarthroplasty using a long femoral stem with distal press-fit fixation, proximal cement augmentation of bone defects, and supplemental plate–cable reconstruction may provide stable reconstruction and support early mobilization and functional recovery in selected patients.

## Strengths and limitations

This case has several strengths. The extensive myeloma-related destruction of the proximal femur represents a challenging reconstructive scenario, and the consistency among preoperative imaging, intraoperative findings, and histopathological confirmation provides a clear basis for the diagnosis and surgical decision-making. The individualized reconstruction strategy, multidisciplinary perioperative management, and 15-month clinical and radiographic follow-up further provide practical information for the management of similar complex cases.

Several limitations should also be acknowledged. First, this is a single case without a comparison group, and therefore the findings cannot establish the superiority of long-stem hemiarthroplasty with proximal cement augmentation over other reconstructive strategies. Second, although the 15-month follow-up provides intermediate-term clinical and radiographic information, longer follow-up is required to evaluate long-term stem fixation, possible biological osseointegration, implant survival, acetabular changes, and progression of the underlying multiple myeloma. Finally, the successful use of intraoperative autologous blood salvage in this patient cannot establish its oncological safety or general applicability in patients with multiple myeloma, and further evidence is required before broader conclusions can be drawn.

## Conclusion

Hemiarthroplasty combined with cemented long-stem reconstruction and supplemental fixation may represent a viable reconstructive option for selected patients with multiple myeloma-related pathological femoral neck fractures and severe proximal femoral osteolysis. Careful assessment of the extent of bone destruction, acetabular involvement, remaining femoral bone stock, systemic disease burden, and functional requirements is essential for individualized surgical decision-making. Multidisciplinary management may further facilitate perioperative planning and postoperative care in medically complex patients.

## Data Availability

The original contributions presented in the study are included in the article/Supplementary Material, further inquiries can be directed to the corresponding author.
