# Supplementary material for: Hemiarthroplasty with a long femoral stem and proximal cement augmentation for a myeloma-related pathological femoral neck fracture: a case report
Source: Front Surg. 2026 Oct 28;13:1819823. doi: 10.3389/fsurg.2026.1819823 (PMC13751958; doi:10.3389/fsurg.2026.1819823)
Supplement: Supplementary file 1 [file Table1.docx]

| **Characteristic** | **Finding** |
| --- | --- |
| Age | 66 years |
| Sex | Female |
| Duration of multiple myeloma | Approximately 1.5 years |
| Major comorbidities | Type 2 diabetes mellitus, poorly controlled hypertension, and anemia |
| Hemoglobin | **106 g/L** |
| Serum creatinine | **91 μmol/L** |
| Serum calcium | **2.27 mmol/L** |
| M-protein | **24.6 g/L** |
| Serum free light chains | **κ: 138.4 mg/L; λ: 13.2 mg/L; κ/λ ratio: 10.48** |
| Disease stage | **ISS stage II; R-ISS stage II** |
| Previous anti-myeloma therapy | **No standardized systemic therapy before the current admission** |

**Supplementary table 1. Baseline clinical characteristics of the patient**
