# Supplementary material for: Hemiarthroplasty with a long femoral stem and proximal cement augmentation for a myeloma-related pathological femoral neck fracture: a case report
Source: Front Surg. 2026 Oct 28;13:1819823. doi: 10.3389/fsurg.2026.1819823 (PMC13751958; doi:10.3389/fsurg.2026.1819823)
Supplement: Supplementary file 2 [file Table2.docx]

**Supplementary table 2. Clinical timeline of the patient**

| **Time** | **Clinical event** |
| --- | --- |
| Approximately 18 months before admission | Multiple myeloma was diagnosed; the disease was classified as ISS stage II and R-ISS stage II. The patient had not received standardized systemic anti-myeloma therapy before the current admission. |
| Day of presentation | The patient sustained a low-energy fall and developed acute right hip pain with inability to bear weight. |
| At admission | Plain radiographs and CT demonstrated a pathological fracture of the right femoral neck with extensive osteolytic destruction of the proximal femur. |
| Preoperative period | Multidisciplinary assessment was performed to evaluate the patient’s hematological disease, medical comorbidities, perioperative risk, and reconstructive strategy. |
| Surgery | Cemented long-stem hemiarthroplasty with cement augmentation and supplemental trochanteric plate/cerclage fixation was performed. |
| Early postoperative period | Pain was substantially relieved, wound healing was satisfactory, and early rehabilitation and assisted ambulation were initiated. Histopathological examination confirmed plasma cell myeloma. |
| At discharge | The patient was discharged in stable condition and referred to the hematology service for subsequent management of multiple myeloma and myeloma-related bone disease. |
| 15 months after surgery | The patient remained ambulatory with satisfactory hip function. Follow-up radiographs showed stable prosthetic and supplemental fixation without major implant-related complications. |
